# The quantification of reproductive hormones in the hair of captive adult brown bears and their application as indicators of sex and reproductive state

**DOI:** 10.1093/conphys/cox032

**Published:** 2017-06-01

**Authors:** Marc Cattet, Gordon B. Stenhouse, David M. Janz, Luciene Kapronczai, Joy Anne Erlenbach, Heiko T. Jansen, O. Lynne Nelson, Charles T. Robbins, John Boulanger

**Affiliations:** 1 RGL Recovery Wildlife Health & Veterinary Services, 415 Mount Allison Crescent, Saskatoon, Saskatchewan, CanadaS7H 4A6; 2 Department of Veterinary Pathology, Western College of Veterinary Medicine, University of Saskatchewan, 52 Campus Drive, Saskatoon, Saskatchewan, CanadaS7N 5B4; 3 fRI Research and Alberta Environment and Parks, 1176 Switzer Drive, Hinton, Alberta, CanadaT7V 1X6; 4 Department of Veterinary Biomedical Sciences, Western College of Veterinary Medicine, University of Saskatchewan, 52 Campus Drive, Saskatoon, Saskatchewan, CanadaS7N 5B4; 5 Toxicology Centre, University of Saskatchewan, 44 Campus Drive, Saskatoon, Saskatchewan, CanadaS7N 5B3; 6 School of the Environment, Washington State University, PO Box 642812, Pullman, WA99164, USA; 7 Department of Integrative Physiology and Neuroscience, College of Veterinary Medicine, Washington State University, 1815 Ferdinand's Lane, Veterinary and Biomedical Research Building 205, Pullman, WA 99164, USA; 8 Department of Veterinary Clinical Sciences, College of Veterinary Medicine, Washington State University, PO Box 647010, Pullman, WA 99164,USA; 9 School of the Environment and School of Biological Sciences, Washington State University, PO Box 644236, Pullman, WA 99164, USA; 10 Integrated Ecological Research, 924 Innes Street, Nelson, British Columbia, CanadaV1L 5T2

**Keywords:** Brown bear, enzyme-linked immunoassay, hair cortisol, hair reproductive hormone profile, non-invasive genetic sampling, *Ursus arctos*

## Abstract

Recognizing the potential value of steroid hormone measurements to augment non-invasive genetic sampling, we developed procedures based on enzyme-linked immunoassays to quantify reproductive steroid hormone concentrations in brown bear (*Ursus arctos*) hair. Then, using 94 hair samples collected from eight captive adult bears over a 2-year period, we evaluated (i) associations between hair concentrations of testosterone, progesterone, estradiol and cortisol; (ii) the effect of collecting by shaving vs. plucking; and (iii) the utility of reproductive hormone profiles to differentiate sex and reproductive state. Sample requirements (125 mg of guard hair) to assay all hormones exceeded amounts typically obtained by non-invasive sampling. Thus, broad application of this approach will require modification of non-invasive techniques to collect larger samples, use of mixed (guard and undercoat) hair samples and/or application of more sensitive laboratory procedures. Concentrations of hormones were highly correlated suggesting their sequestration in hair reflects underlying physiological processes. Marked changes in hair hormone levels during the quiescent phase of the hair cycle, coupled with the finding that progesterone concentrations, and their association with testosterone levels, differed markedly between plucked and shaved hair samples, suggests steroids sequestered in hair were likely derived from various sources, including skin. Changes in hair hormone concentrations over time, and in conjunction with key reproductive events, were similar to what has been reported concerning hormonal changes in the blood serum of brown bears. Thus, potential for the measurement of hair reproductive hormone levels to augment non-invasive genetic sampling appears compelling. Nonetheless, we are conducting additional validation studies on hair collected from free-ranging bears, representative of all sex, age and reproductive classes, to fully evaluate the utility of this approach for brown bear conservation and research.

## Teaser

We used enzyme immunoassays to measure testosterone, progesterone, estradiol and cortisol concentrations in serial hair samples collected from captive adult brown bears. Reproductive hormone levels changed throughout the year in step with different reproductive events underscoring the strong potential for these measurement to be used to augment non-invasive genetic sampling.

## Introduction

The refinement of DNA amplification techniques and their use for the identification of species, sex and individual animals from hair samples has had an immense impact on wildlife management, conservation and research in the past few decades. It is now possible, and becoming common practice, to estimate population features and processes, such as abundance (Woods *et al*., 1999; Kendall *et al*. 2009), genetic diversity and structure (Proctor *et al*., 2010; Schregel *et al*., 2015), relatedness (Ritland, 1996; Zedrosser *et al*., 2007), gene flow (Taberlet *et al*., 1997; Kopatz *et al*., 2014) and response to translocation (De Barba *et al*., 2010; Mukesh *et al*., 2015) without live capture through the use of various hair collection techniques, collectively termed non-invasive hair sampling (Kendall and McKelvey, 2008). Relative to live capture, non-invasive hair sampling, or more generally, non-invasive genetic sampling (i.e. hair or faeces), offers a viable alternative approach that can increase sampling success, reduce sampling cost and increase animal and field personnel safety (MacKay *et al*., 2008; Mumma *et al*., 2015).

Information provided through DNA analysis is limited though, and does not provide insight regarding age, body condition, reproductive status and other types of information typically collected under live capture programmes. This has prompted research to identify physiological indicators that can be reliably quantified from samples collected noninvasively to provide insight into the health and fitness of individuals, and into the performance of their populations. In this regard, a considerable amount of research has been published in recent years focused on the measurement of steroid hormones, that include cortisol, testosterone, progesterone and estradiol, in hair samples collected from various wild mammals. Most of this research though has been directed toward using hair cortisol levels to assess the hypothalamic–pituitary–adrenal response over time to natural and human-caused stressors that include, but are not limited to, inter- and intra-specific resource competition (Bryan *et al*., 2014a; Lafferty *et al*., 2015), maternal fetal programming (Kapoor *et al*., 2016; Meise *et al*., 2016), hunting intensity (Bryan *et al*., 2014b), contaminant exposure (Bechshøft *et al*., 2015) and anthropogenic disturbance (Agnew *et al*., 2016; Carlitz *et al*., 2016), including climate change (Macbeth *et al*., 2012; Mislan *et al*., 2016). However, to date, little research has been directed toward evaluating concurrent concentrations of multiple steroid hormones (termed steroid hormone profiles) in hair for their potential value in augmenting non-invasive genetic sampling (Schwartz and Monfort, 2008).

The application of hair steroid hormone profiles in wildlife management, conservation and research must be supported by requisite validation studies (Heistermann, 2010; Fourie *et al*., 2016). Otherwise, the results and conclusions drawn from such usages are uncertain. Validation comprises numerous types of studies. These typically include initial laboratory studies to evaluate the effects of different storage, washing and drying methods on sample and physiological indicator integrity (Macbeth *et al*., 2010; Yamanashi *et al*., 2016; Kroshko *et al*., 2017), and to establish performance specifications for the physiological indicator assay(s) such as the reportable range, analytical sensitivity, precision and accuracy (Lee *et al*., 2006; Burd, 2010). Validation efforts should also include studies of animals under captive situations (e.g. zoos) or in the field to identify factors that may confound interpretation of the physiological indicator of interest (Cattet *et al*., 2014; Carlitz *et al*., 2015; Salaberger *et al*., 2016) and to demonstrate that it is sensitive to the physiological process of interest (Terwissen *et al*., 2014; Carlitz *et al*., 2016).

In this validation study, we evaluated the measurement of three steroid hormones—testosterone, progesterone and estradiol—in hair samples collected from captive adult brown bears (*Ursus arctos*) to determine the potential value of the hair reproductive hormone profile in augmenting non-invasive genetic sampling by providing information on sex and reproductive state. Our interest in reproduction is because of its importance as an attribute of health (i.e. reproduction may be suppressed or cease when health is compromised) and biological fitness in individual animals (Eberhardt, 2002; Zedrosser *et al*., 2013), and because it is required as a measurement to estimate reproductive rates at the population level (Garshelis *et al*., 2005). Our specific research objectives were to:

Develop laboratory procedures and establish performance specifications to quantify reproductive steroid hormone (testosterone, progesterone, estradiol) concentrations in brown bear hair.
Determine if the hair concentrations of reproductive hormones, as well as cortisol, another steroid hormone, are correlated with each other.
Evaluate if the method of hair collection, plucked which provides samples that include follicles, or shaved which provides samples composed only of hair shafts, is related to the concentrations of reproductive hair hormones in a consistent manner (e.g. consistently higher with one method vs. the other).
Establish if the hair reproductive hormone profile of captive adult brown bears differs between sexes and/or changes between different times of the year (hibernation, pre-breeding, breeding, post-breeding).

## Methods

### Sources of brown bear hair

We used hair samples collected from two free-ranging brown bears that were killed in response to human-wildlife conflicts in Alberta, Canada, and from eight captive brown bears housed at the Washington State University (WSU) Bear Research, Education and Conservation Centre. The full hides from the killed bears, an immature (<5 years) male and female, were removed and frozen immediately following death. We acquired the frozen hides from Alberta Environment and Parks’ Fish and Wildlife Division as ‘limitless sources’ of hair to address the first objective of this study to develop accurate and reliable laboratory procedures to measure testosterone, progesterone and estradiol concentrations in hair samples in the amounts that might be obtained by using non-invasive techniques, e.g. 30 mg by a single barbed wire snag or upwards of 150 mg when using closely-spaced, multiple barbs.

For the remaining objectives, we used 94 hair samples (~125 mg per sample) collected at WSU over a 2-year period from April 2013 to April 2015. Although the collection of hair samples was incidental to other research, details regarding the handling and sampling of bears at WSU have been reported by Joyce-Zuniga *et al*. (2016). Thirteen samples per bear were collected from two adult females (ranging from 10.8 to 12.3 years over the study period) that bred in May 2014, gave birth to two cubs each in January 2015, and were lactating when final hair samples were collected in April 2015. Overall, 6–14 samples per bear were collected from four adult females (8.3–10.1 years over the study period) that were administered megestrol acetate (orally at 40–160 mg/day) as a method of birth control from mid-April until the end of June in both years. Overall, 12–13 samples per bear were collected from two adult males (11.8–13.1 years over the study period) that bred in May 2015. Of the 94 samples, 64 were collected using electric clippers to sever hair shafts at their point of emergence from the skin (shaved samples). Thirty samples were collected by plucking the hair from the skin so that the sample contained many hairs with intact follicles (plucked samples). Twenty of these were collected as paired samples where hair was collected by both shaving and plucking from the same location, on the same bear, at the same time. The purpose of using two collection methods was to allow us to evaluate if the presence or absence of follicles significantly affected reproductive hormone profiles. All samples were collected from the top of the shoulders, placed in paper envelopes that were left open for several hours to ensure that the hair was air-dried, then sealed and stored under low light at room temperature (~20°C) until the analysis of hormone levels in the following 8–16 months. Although the long-term stability of reproductive hormones in hair has yet to be determined, we have no reason to believe that long-term stability was an issue on the basis that the concentration of cortisol has been demonstrated to remain stable in brown bear hair for at least 17 months (Macbeth *et al*., 2010), and in chimpanzee (*Pan troglodytes*) hair for at least 2 years (Yamanashi *et al*., 2016). Further, cortisol has been extracted from archaeological human hair samples, dating as far back as AD550, and levels were found to be comparable to the range of values measured in hair collected from modern individuals (Thomson, 2008; Webb *et al*., 2010, 2015).

The captive bears at WSU are maintained according to the Bear Care and Colony Health Standard Operating Procedures (Protocol #04 873) approved by the Washington State Institutional Animal Care and Use Committee, and based on U.S. Department of Agriculture guidelines.

### Sample preparation and handling

Sample selection, removal of surface contaminants and sample preparation for hormone extraction were performed according to the hair cortisol protocol developed by Macbeth *et al*. (2010). Guard hairs were preferentially selected for analysis for all animals. Gross contaminants such as mud, dried faeces and plant matter were removed with fine forceps and gentle tapping of the hair sample cushioned between two paper towels. Extreme care was taken to prevent damage to hair shafts. The mass of intact hair required (~125 mg) to measure all four hormones (testosterone, progesterone, estradiol and cortisol) was prepared for extraction as a single batch of hair to ensure minimal loss of hair and identical handling of each sample. Hair samples were washed three times in 0.04 mL of methanol/mg of hair for 3 min per wash on a slow (12 revolutions/min) end-over-end rotator. Hair was removed from the methanol after each wash and gently blotted dry. Fresh methanol was used for each wash cycle. Decontaminated hair was placed in a plastic petri dish lined with filter paper with the lid off set to allow for airflow and allowed to dry at ambient temperature for 3 days. Dry decontaminated hair was ground to a consistent fine powder using a Retsch MM 301 mixer mill (Retsch Inc, Newtown, PA, USA). Samples were ground for 0.03 min/mg of hair at 30 Hz in a 10 mL stainless steel grind jar with one 12 mm stainless steel grinding ball. Powdered hair was collected and transferred to a 1.5 mL tube and stored in the dark at room temperature prior to hormone extraction.

### Hormone extraction and analysis

The cortisol extraction protocol developed by Macbeth *et al*. (2010) was used to extract cortisol, progesterone and testosterone. Briefly, 0.5 mL of HPLC grade methanol was added to each ground hair sample. Samples were mixed with a vortex mixer for 10 s and placed in a slow end-over-end rotator at room temperature for 24 h. After 24 h, samples were centrifuged at 4500 rpm for 15 min at 20°C. Supernatants were collected into 12 × 75 mm^2^ glass culture tubes, and solvent was evaporated under a gentle stream of nitrogen gas. The extracted powder was rinsed with another 0.5 mL of methanol, mixed with a vortex mixer for 40 s, centrifuged, the supernatant collected and evaporated as before. Extracts were concentrated to the bottoms of the tubes using consecutive rinses of methanol in decreasing volumes (0.4, 0.2, 0.15 mL) and were dried under nitrogen gas after each rinse.

For estradiol extraction, 10 mL of methyl tert-butyl ether (EMD Chemicals, Gibbstown, NJ, USA) was added to each 50 mg powdered hair sample in a 16 × 125 mm^2^ glass culture tube. Samples were mixed with a vortex mixer for 10 s and placed in slow rotator for 24 h. Samples were then centrifuged, supernatants collected and dried under nitrogen gas as described previously.

Extracted hormones were reconstituted in 125–250 μL of the buffer provided by the respective hormone kits. Samples were left to reconstitute for 12 h in the dark at 4°C then gently mixed, centrifuged at 4500 rpm for 5 min at 20°C, and the supernatant collected and stored at −20°C until analysis.

Hormone concentrations were determined in duplicate using commercially available enzyme-linked immunosorbent assays (ELISA) specific to each hormone. ELISA kits were chosen based on their sensitivity (i.e. lowest standard) and the volume of sample required to run in duplicate (due to the small volumes of reconstituted hormone extracts obtained). Cortisol kits were purchased from Oxford Biomedical (Rochester Hills, MI, USA), progesterone and testosterone kits were from Enzo Life Sciences (Ann Arbor, MI, USA), and estradiol kits were from Calbiotech (Spring Valley, CA, USA). Samples were diluted as required (progesterone 1/10, testosterone 1/5, cortisol and estradiol undiluted) with the appropriate buffer provided with each kit before being assayed.

### Statistical analysis

Assay performance specifications—we calculated the lower limits of detection (LOD) for the testosterone, progesterone and estradiol assays using the following formula:

$$\mathrm{LOD}=\left({\mathrm{2SD}}_{\mathrm{B0}}/\Delta \text{OD}\right)\times \mathrm{lowest standard concentration},$$

where SD_B0_ was the standard deviation of the zero standard (B0), and ΔOD was the change in between the zero standard and the lowest concentration standard.

We assessed parallelism between serially diluted samples and standards provided with each kit using the Curve Estimation procedure in IBM SPSS Statistics (Version 23; IBM Corporation, Armonk, NY, USA). Assessments were based on triplicate runs for each assay.

To measure extraction efficiency, we added 0.05 mL of a 0.54 ng/mL testosterone standard (≥98% purity, Sigma-Aldrich, Munich, Germany), and 0.05 mL of a 2.61 ng/mL progesterone standard (≥99% purity, Sigma-Aldrich, Munich, Germany), to 25 mg samples of hormone-stripped powdered hair (three samples for each hormone).

**Figure 1:**
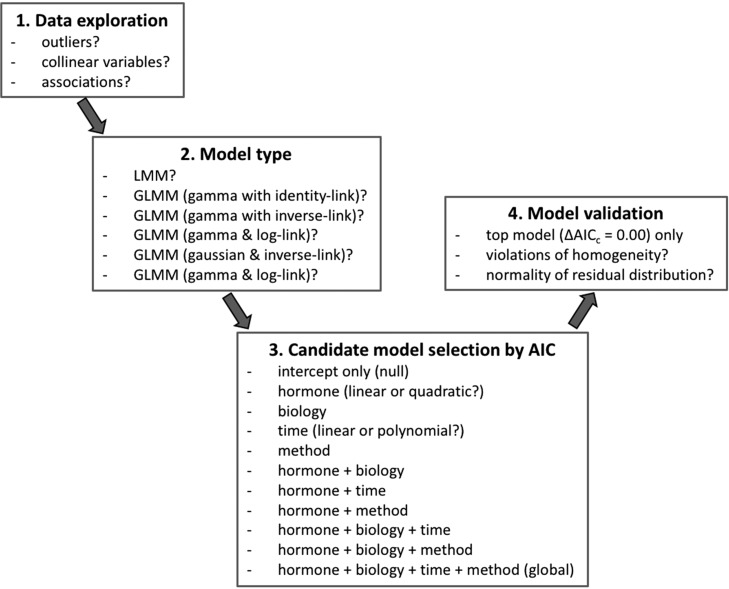
Sequence of procedures followed in the statistical analysis of reproductive hormone concentrations measured in 94 hair samples collected from April 2013 to April 2015 from eight captive adult brown bears housed at the WSU Bear Research, Education and Conservation Centre. Interaction terms are not provided in the candidate models shown in the figure, but were included in the analyses and are discussed in the text. Abbreviations used are linear mixed models (LMM), generalized linear mixed models (GLMM), Akaike information criterion (AIC), and difference in sample-size–adjusted Akaike information criterion (AIC_C_) values between top model and another candidate model (∆AIC_C_).

Hair hormone concentrations in captive adult brown bears—using R 3.3.1 (R Core Team, 2016), we analyzed hair hormone values determined for 94 hair samples following the steps outlined in Fig. 1. We used a univariate approach where the hair concentration (pg/mg) for each of the three reproductive hormones was the response variable for a single path of analysis. Each analysis was based on the same fixed effects and several two-way interactions, with bear identification included as a random effect (intercept only) (Table 1). Although we also considered using hormone ratios to analyze the simultaneous effects of two hormones, the interpretation of hormone ratios can be complicated, in part, because of the asymmetry caused by differences in distribution between the numerator and denominator hormones (Sollberger and Ehlert, 2016). Further, use of hormone ratios limits the evaluation of interaction effects that may be unique to the numerator and/or denominator hormone.

**Table 1:** Variables evaluated as potential determinants of the reproductive hormone concentrations in 94 hair samples collected from April 2013 to April 2015 from eight adult brown bears housed at the Washington State University (WSU) Bear Research, Education and Conservation Centre

Category	Variable	Variable type	Variable categories or range of values
*1) Fixed effects*			
Hormone	Reproductive hormone (pg/mg of hair)	Continuous	Testosterone (0.8–25.0), progesterone (1.0–16.6) or estradiol (0.0041–0.0306)
	Cortisol (pg/mg of hair)	Continuous	0.13–2.46
Biology	Sex and reproductive class	Categorical	Breeding female, non-breeding female or breeding male
	Age (years)	Continuous	8–13
Time	Physiological phase	Categorical	Hibernation (October 20 to March 20), pre-breeding (March 21 to April 25), breeding (April 26 to June 20) or post-breeding (June 21 to October 19)
	Adjusted ordinal day	Discrete	1–365 with March 21 set as Day 1
Method	Follicle removed prior to analysis	Categorical	Yes or no
*2) Random effect*			
	Bear identification	Categorical	Unique for each bear

Dates for physiological phases obtained from Robbins *et al*. (2012) and personal communication by Joy Anne Erlenbach.

For data exploration, we followed the protocol described in Zuur *et al*. (2010): (i) Cleveland dot-plots were used to evaluate variables for potential outliers, (ii) pair-plots (Pearson *r* ≥ 0.70) and generalized variance inflation factors (GVIF^1/(2·df)^ ≥ 3.0) were used to identify collinear variables, (iii) multi-panel scatterplots were used to visualize associations between response variables (reproductive hormone concentrations) and continuous predictor variables (Table 1) and (iv) multi-panel boxplots were used to visualize associations between response variables and categorical predictor variables (Table 1). For this step, we made extensive use of the ‘lattice’ package (Sarkar, 2008) in R, as well as the custom R code provided by Ieno and Zuur (2015).

Prior to model development, we standardized all continuous predictor variables by subtracting the mean from the individual observed values and then dividing by the standard deviation. The use of standardized variables can be helpful in interpreting model coefficients, especially when variables range in value over different scales, e.g. testosterone vs. estradiol. To determine an appropriate model type, we used an Information Theoretic approach to compare six random intercept model types, as outlined in Fig. 1, by Aikaike Information Criteria (AIC) weights (Burnham and Anderson, 2002). We constructed generalized linear mixed models (GLMM) using the ‘glmer’ function in package ‘lme4’ (Bates *et al*., 2015). We calculated AIC weights using the ‘aictab’ function in package ‘AICcmodavg’ (Mazerolle, 2016) for models composed of all fixed effects, but no interactions, and selected the model type with the highest Akaike weight.

We constructed 11 candidate models, including intercept only (null) and global models, as listed in Fig. 1. For the hormone and time models, we first determined whether to use a linear or a quadratic association between response and predictor variables (hormone vs. hormone^2^, adjusted ordinal day vs. adjusted ordinal day^2^ vs. adjusted ordinal day^3^) by selecting the model with the highest Akaike weight. This model was then used as a candidate model. It was also used to construct candidate models with two or more categories, e.g. hormone + biology, hormone + biology + time. Although not shown in Fig. 1, many of the candidate models also included interaction terms that, in our opinion, represented reasonable biological possibilities. We calculated a coefficient of determination based on the likelihood-ratio test (*R*^2^_LR_) for each model using the ‘r.squaredLR’ function in package ‘MuMIn’ (Bartoń, 2016). Data were complete for all 94 records, i.e. no missing values. Consequently, we proceeded with comparing among all models using AIC weights, and then we selected the top model (∆AICc = 0.00) for further analysis. However, we also considered other models where ∆AICc ≤ 2.00.

We used the ‘R2jags’ function (Su and Yajima, 2015) in package ‘JAGS’ (Plummer, 2003) to resample our data and improve the precision of coefficient means and 95% Bayesian credible intervals (BCI). Five chains were used in the Markov Chain Monte Carlo (MCMC) process with a burn-in of 40 000 iterations, a thinning rate of 10, and 50 000 iterations for each posterior distribution. We visually assessed the mixing of chains for each parameter using the ‘MyBUGSChains’ function in package ‘JAGS’ (Plummer, 2003). The standardized coefficient means and 95% BCI presented within the tables and figures of this report are those that were derived through the MCMC process, not those that were derived through the initial model development process.

To determine if top models (one for each hormone) were valid, we first calculated Pearson residuals using the ‘resid’ function in package ‘stats’ (R Core Team, 2016). We then plotted them against fitted values predicted by the top models, and against standardized values of all continuous explanatory variables irrespective of whether or not they were included in the top model, to identify violations of homogeneity (patterns in the residuals and/or lack of fit) and influential observations. We also used quantile–quantile (q–q) plots to assess normality in the distributions of residuals. We developed figures to help interpret significant interactions using the ‘ggplot2’ package (Wickham, 2009). We considered parameters as ‘statistically significant’ when their 95% BCI did not include a value of 0. In other words, the range of BCI values were either greater than, or less than, zero.

## Results

### Assay performance specifications

The LOD for testosterone, progesterone and estradiol were 6.71, 4.11 and 1.39 pg/mL, respectively. Serially diluted extracts were parallel with standards for testosterone (*R*^2^ = 0.983, *P* < 0.001), progesterone (*R*^2^ = 0.991, *P* < 0.001) and estradiol (*R*^2^ = 0.978, *P* < 0.001). The intra-assay coefficient of variation (%CV) was 4.7% for testosterone (*N* = 6), 2.3% for progesterone (*N* = 5) and 6.8% for estradiol (*N* = 6). The inter-assay %CV was 10.5% for testosterone (*N* = 12), 10.8% for progesterone (*N* = 10) and 10.5% for estradiol (*N* = 12). Extraction efficiency (mean ± SD) of spiked samples was 116.0 ± 3.7% for testosterone and 111.0 ± 7.6% for progesterone. We could not reliably determine the extraction efficiency for estradiol; repeated attempts resulted in unrealistically high values that ranged from 161 to 198%. The performance specifications for the hair cortisol assay were reported previously (Macbeth *et al*., 2010).

### Hair hormone concentrations in captive adult brown bears

#### Observed values

**Figure 2:**
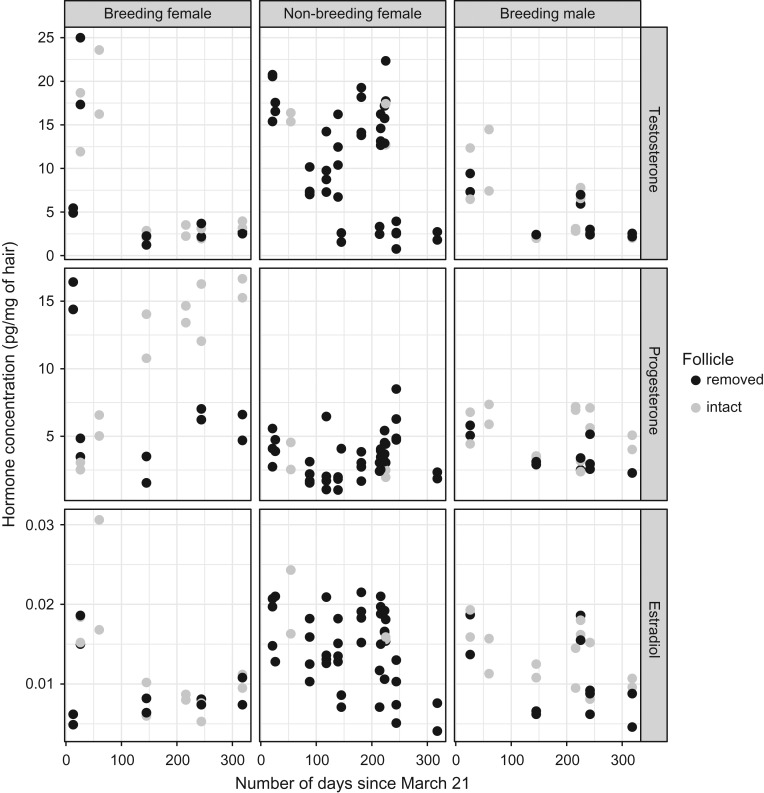
Reproductive hormone concentrations measured in 94 hair samples collected from eight adult captive brown bears between April 11, 2013 (Day 21) and April 3, 2015 (Day 13). Twenty-eight samples were collected from two adult females (11.3–12.3 years) that bred in May 2014 (41–71 days since March 21st) and gave birth to four cubs in early to mid-January 2015 (285–300 days). Forty-one samples were collected from four adult females (8.3–10.8 years) that were administered megestrol acetate (orally at 40–160 mg/day) as a method of birth control from April 20 to June 19 (30–90 days) in each year. Twenty-five samples were collected from two adult males (11.8–13.1 years) that bred in May 2014. Sixty-four hair samples were collected using electric clippers to sever the hair shaft at the skin surface, thus removing the follicle. At 30 sampling times, a sample was collected by plucking hair (follicle intact) with a hemostatic clamp from the skin. At 20 of these sampling times, hair was collected as paired samples by both shaving and plucking from the skin at adjacent body locations. Physiological phases correspond with: (i) pre-breeding—Days 1–35; (ii) breeding—Days 36–91; (iii) post-breeding—Days 92–212; and (iv) hibernation—Days 213–365.

Hair hormone concentrations varied widely among bears (Fig. 2), including between sex and reproductive classes (Table 2). Irrespective of method of hair collection, mean and maximum testosterone values were greater in the female classes than in males, and mean and maximum progesterone values were greater in breeding females than in non-breeding females and breeding males. No clear trends were evident with estradiol or cortisol values.

**Table 2:** Steroid hormone concentrations^a^ in 94 hair samples collected from April 2013 to April 2015 from eight adult brown bears housed at the Washington State University (WSU) Bear Research, Education and Conservation Centre

Method of collection	Hormone (pg/mg of hair)	Sex and reproductive class [number of individuals]		
		Breeding female [2]	Non-breeding female [4]	Breeding male [2]
Plucked [includes follicle]	Testosterone	8.81 (14) [1.94–23.59]	15.88 (2) [15.37–16.38]	5.32 (14) [1.99–14.47]
	Progesterone	9.62 (17) [1.96–16.66]	3.54 (2) [2.54–4.55]	5.21 (14) [2.38–7.36]
	Estradiol	0.013 (17) [0.005–0.031]	0.020 (2) [0.016–0.024]	0.013 (14) [0.008–0.019]
	Cortisol	1.09 (17) [0.43–2.16]	0.36 (2) [0.32–0.40]	1.07 (14) [0.46–1.94]
Shaved [no follicle]	Testosterone	8.01 (14) [1.22–25.00]	10.71 (39) [0.76–20.77]	4.30 (11) [2.17–9.42]
	Progesterone	6.90 (14) [1.55–16.42]	3.38 (39) [1.02–8.50]	3.22 (11) [2.28–5.81]
	Estradiol	0.011 (14) [0.005–0.019]	0.014 (39) [0.004–0.022]	0.011 (11) [0.005–0.019]
	Cortisol	1.18 (14) [0.63–2.79]	0.68 (39) [0.13–2.46]	1.11 (11) [0.57–1.56]

^a^Hair hormone concentrations are presented as mean value, number of hair samples in round brackets, and range from minimum to maximum values in square brackets.

Hair hormone concentrations appeared similar between plucked and shaved hair samples in Table 2. However, comparison of hormone levels derived from paired samples (*N* = 20 pairs) collected at the same body location, from the same bear, at the same time, suggested that method of hair collection (plucked vs. shaved) did, in fact, affect hormone concentrations. Specifically, the mean progesterone concentration was greater (paired *t*-test: *t* = 3.078, *P* = 0.006) in plucked than shaved samples (mean ± SE: 7.03 ± 1.147 pg/mg vs. 3.91 ± 0.354 pg/mg). Mean estradiol (0.013 ± 0.0009 pg/mg vs. 0.011 ± 0.0011 pg/mg) and cortisol levels (1.16 ± 0.101 pg/mg vs. 1.06 ± 0.078 pg/mg) were also higher, albeit not statistically significant (estradiol: *t* = 2.073, *P* = 0.052; cortisol: *t* = 1.844, *P* = 0.081), in plucked samples whereas mean testosterone values were similar (*t* = 1.548, *P* = 0.138) between sample types (6.22 ± 1.216 pg/mg vs. 7.08 ± 1.653 pg/mg). Among paired samples, the correlation in hormone levels between sample types was positive and linear for all four hormones (Pearson correlation coefficients: testosterone—*r* = 0.97, *P* ≤ 0.001; progesterone—*r* = 0.51, *P* = 0.022; estradiol—*r* = 0.80, *P* ≤ 0.001; and cortisol—*r* = 0.85, *P* ≤ 0.001).

Hormone levels also varied markedly within individual bears with maximum ranges in serial samples as follows: (i) 2.2–25.0 pg/mg for testosterone (*N* = 5 shaved samples for one individual), (ii) 1.9–16.7 pg/mg for progesterone (*N* = 7 plucked samples for one individual) and (iii) 0.0076–0.0243 pg/mg for estradiol (*N* = 7 plucked samples for one individual).

Observed hormone concentrations within sex and reproductive classes also varied differentially by physiological phase (Fig. 2). For example, testosterone concentrations during pre-breeding (Days 1–35) and breeding (Days 36–91) phases were higher in females than males, whereas progesterone concentrations during hibernation (Days 213–365) were highest in breeding females. Estradiol concentrations appeared similar across sex and reproductive classes throughout the year. However, sample size was a limiting factor such that many sex and reproductive classes by physiological phase groupings were represented by fewer than ten hair samples (Table S1—Note that herein table or figure numbers preceded by ‘S’ refer to results presented in the Supplementary materials).

#### MCMC diagnostics and model validation

We determined that a GLMM, with a gamma distribution and log-link function, was the best approach to estimating the expected values of response variables (testosterone, progesterone and estradiol) as a function of the predictor variables in the various candidate models shown in Tables S2a–S4a. When resampling the top models presented in these tables, the MCMC chains mixed well and converged to the same posterior distributions for all fixed effects, including two-way interactions (Figs S1–S3). We did not find any trends in the scatterplots of Pearson residuals vs. fitted values, nor any glaring deviations from normality in the distributions of the residuals (Figs S1–S3). Additionally, in plots of Pearson residuals against continuous variable values, the resultant points appeared to be distributed uniformly around the zero-axis (results not shown).

#### Testosterone

The fixed effects for the top model (T1) explained ~82% of the variation in hair testosterone concentrations among bears (Table S2a). The 95% BCI (0.1509–0.9526) for bear identification did not include a value of zero which supported its inclusion as a random intercept in the model. The next ranked model, T2, was not supported by our analysis (∆AIC_C_ = 2.35, *w*_*i*_ = 0.24). Testosterone concentrations were significantly related (i.e. 95% BCI for posterior mean coefficient did not include zero) to cortisol, estradiol, sex and reproductive class and ordinal day (Table S2b). In addition, the association between testosterone and estradiol was significantly related to sex and reproductive class.

**Figure 3:**
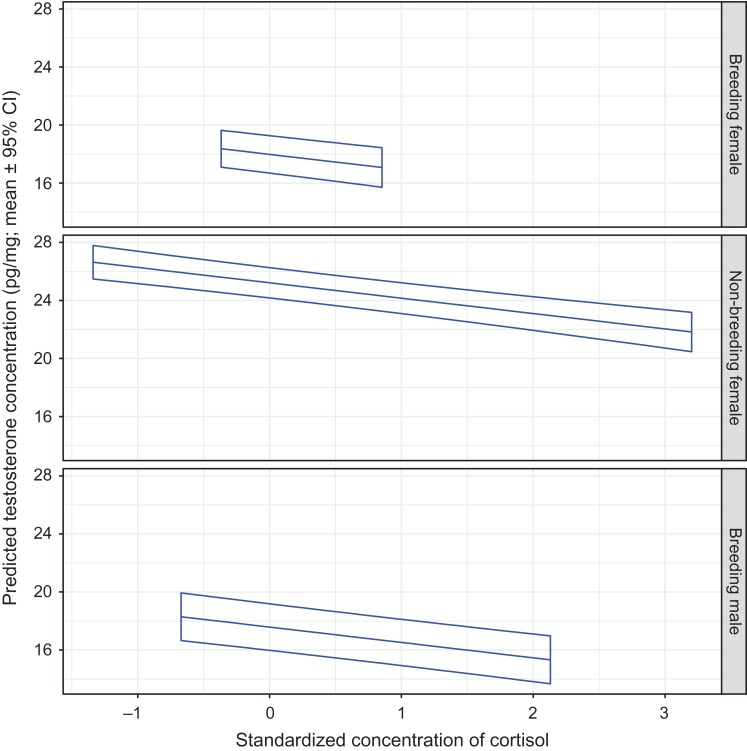
The predicted mean testosterone concentration in the hair of captive adult brown bears in relation to the standardized hair cortisol concentration, and by sex and reproductive class. The means and 95% confidence intervals were estimated by resampling of data from 94 records using model T1 presented in Table S2a. The analysis was constrained to bears that were sampled during the hibernation phase on January 4th. Standardized continuous variables in model T1 were set at mean values as follows: estradiol = 0, progesterone = 0 and ordinal day = 1.306.

**Figure 4:**
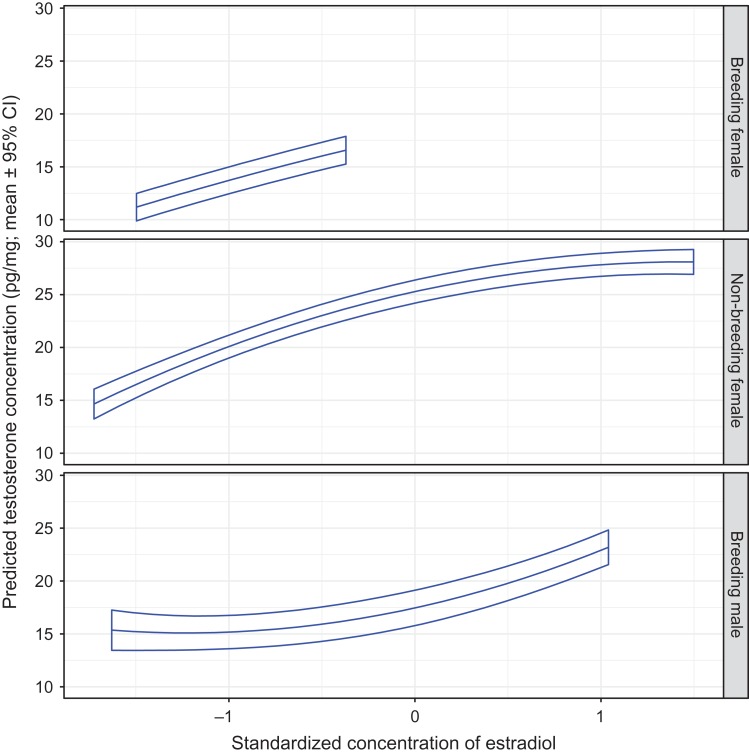
The predicted mean testosterone concentration in the hair of captive adult brown bears in relation to the standardized hair estradiol concentration, and by sex and reproductive class. The means and 95% confidence intervals were estimated by resampling of data from 94 records using model T1 presented in Table S2a. The analysis was constrained to bears that were sampled during the hibernation phase on January 4th. Standardized continuous variables in model T1 were set at mean values as follows: cortisol = 0, progesterone = 0 and ordinal day = 1.306.

The hair testosterone concentration in all bears decreased as the hair cortisol concentration increased (Fig. 3). Conversely, hair testosterone increased as hair estradiol increased (Fig. 4). When holding cortisol or estradiol values constant, testosterone levels were ~50% greater in non-breeding females than in breeding females or breeding males. As hair estradiol levels increased, the slope of increase in the testosterone levels for non-breeding females decreased (i.e. plateaued) whereas the slope of increase for males increased (i.e. steepened) (Fig. 4).

**Figure 5:**
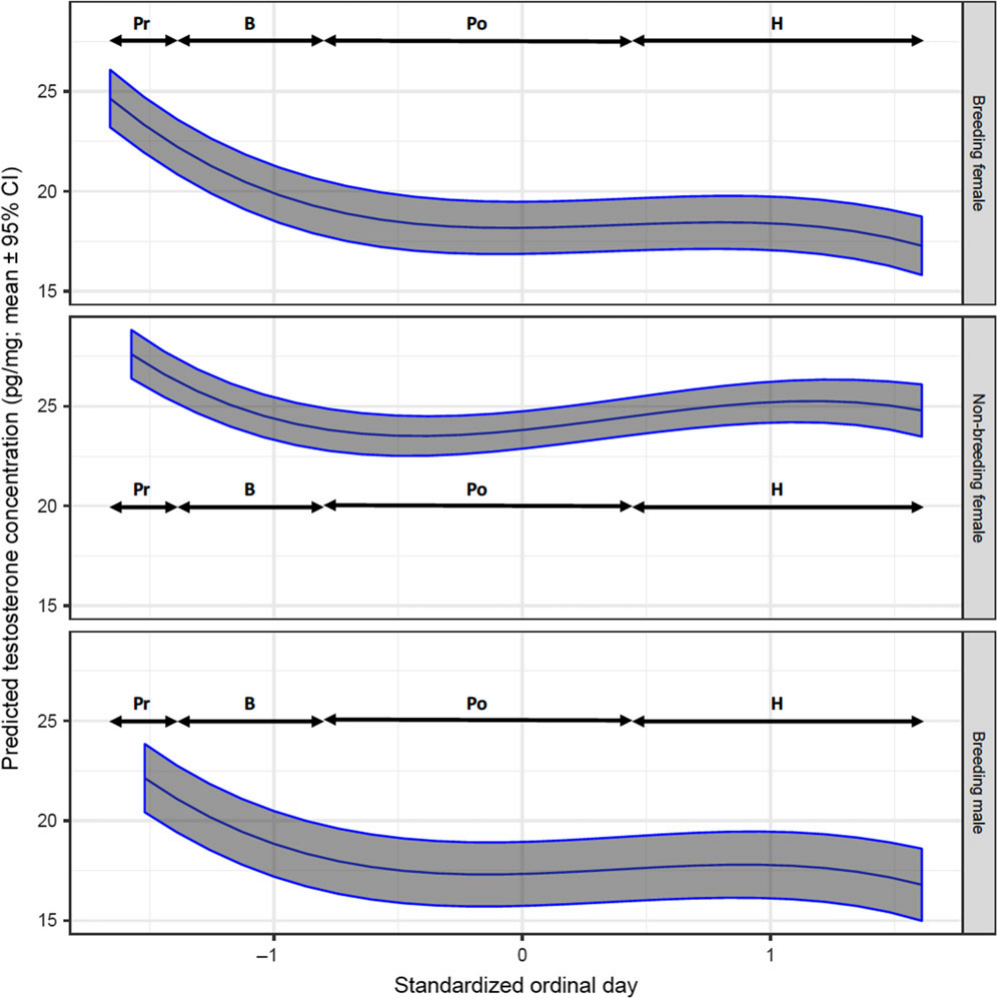
The predicted mean testosterone concentration in the hair of captive adult brown bears in relation to the standardized ordinal day of sampling, and by sex and reproductive class. The means and 95% confidence intervals were estimated by resampling of data from 94 records using model T1 presented in Table S2a. Physiological phases correspond with standardized ordinal day ranges as follows: pre-breeding (Pr: −1.660 to −1.424), breeding (B: −1.414 to −0.824), post-breeding (Po: −0.813 to 0.474) and hibernation (H: 0.484–1.610). Standardized continuous variables in model T1 were set at mean values as follows: cortisol = 0, estradiol = 0 and progesterone = 0.

In all bears, hair testosterone levels were greatest during pre-breeding based on the analysis of shaved samples (Fig. 12), or at the onset of breeding based on the analysis of plucked samples (Fig. 11). Irrespective of sample type (shaved or plucked) though, testosterone levels decreased throughout the breeding phase and early post-breeding reaching lowest levels by August, and remaining at low levels throughout post-breeding and hibernation phases (Figs 5, 11 and 12).

#### Progesterone

The fixed effects for the top model (P1) explained ~66% of the variation in hair progesterone concentrations among bears (Table S3a). The 95% BCI (0.0177–0.4783) for bear identification did not include a value of zero which supported its inclusion as a random intercept in the model. The next ranked model, P2, was not supported by our analysis (∆AIC_C_ = 7.49, *w*_*i*_ = 0.02). Progesterone concentrations were significantly related to cortisol, sex and reproductive class, ordinal day and method of hair collection (Table S3b). Further, the association between progesterone and ordinal day was significantly related to sex and reproductive class, and the associations between progesterone and cortisol, and between progesterone and testosterone, were related to method of hair collection.

**Figure 6:**
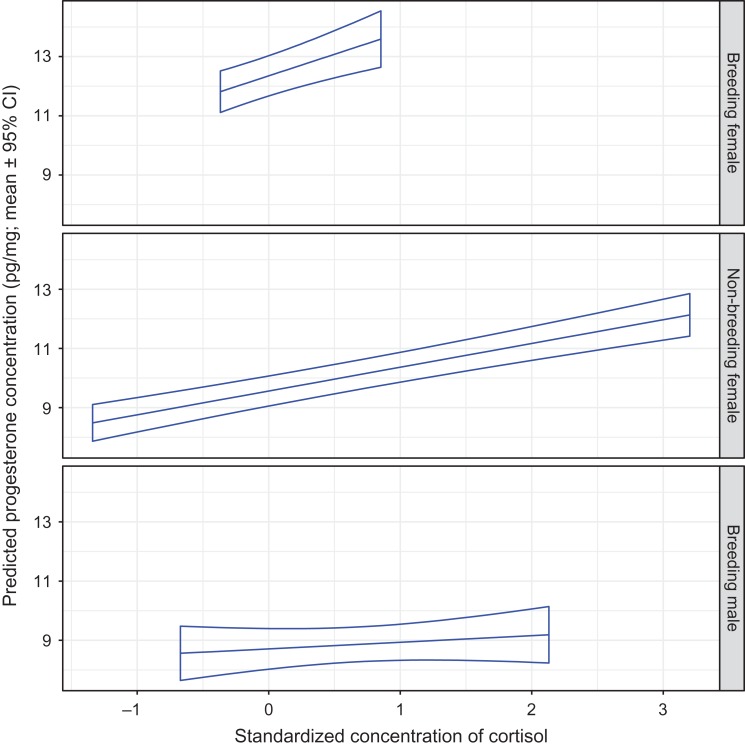
The predicted mean progesterone concentration in the hair of captive adult brown bears in relation to the standardized hair cortisol concentration, and by sex and reproductive class. The means and 95% confidence intervals were estimated by resampling of data from 94 records using model P1 presented in Table S3a. The analysis was constrained to bears that were sampled by shaving hair (no follicles) during the hibernation phase on January 4th. Standardized continuous variables in model P1 were set at mean values as follows: testosterone = 0, estradiol = 0 and ordinal day = 1.306.

The hair progesterone concentration in all bears increased as the hair cortisol concentration increased (Fig. 6). When holding cortisol values constant, progesterone levels were ~30% greater in breeding females than in non-breeding females or breeding males.

**Figure 7:**
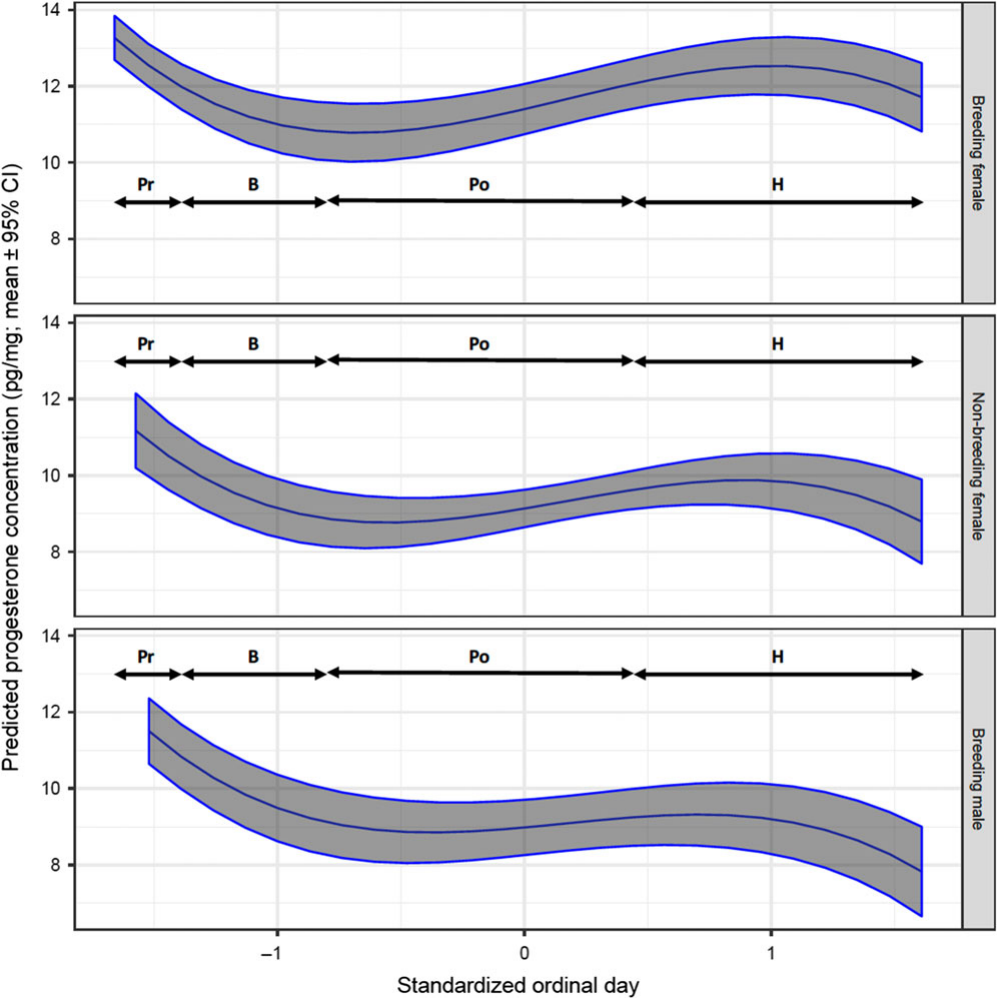
The predicted mean progesterone concentration in the hair of captive adult brown bears in relation to the standardized ordinal day of sampling, and by sex and reproductive class. The means and 95% confidence intervals were estimated by resampling of data from 94 records using model P1 presented in Table S3a. The analysis was constrained to bears that were sampled by shaving hair (no follicles). Physiological phases correspond with standardized ordinal day ranges as follows: pre-breeding (Pr: −1.660 to −1.424), breeding (B: −1.414 to −0.824), post-breeding (Po: −0.813 to 0.474) and hibernation (H: 0.484–1.610). Standardized continuous variables in model T1 were set at mean values as follows: cortisol = 0, testosterone = 0 and estradiol = 0.

Progesterone levels in non-breeding females and breeding males fluctuated over a small range of ~4 pg/mg throughout the year with lowest values tending to occur during hibernation (Figs 7, 11 and 12). In breeding females, however, progesterone levels increased following breeding, and continued to increase into hibernation. The progesterone increase was most evident in the hair samples plucked from breeding females (Fig. 11) where the increase was sustained from pre-breeding to hibernation. In contrast, the progesterone levels in hair samples collected by shaving did not increase noticeably until the latter half of hibernation (Fig. 12).

**Figure 8:**
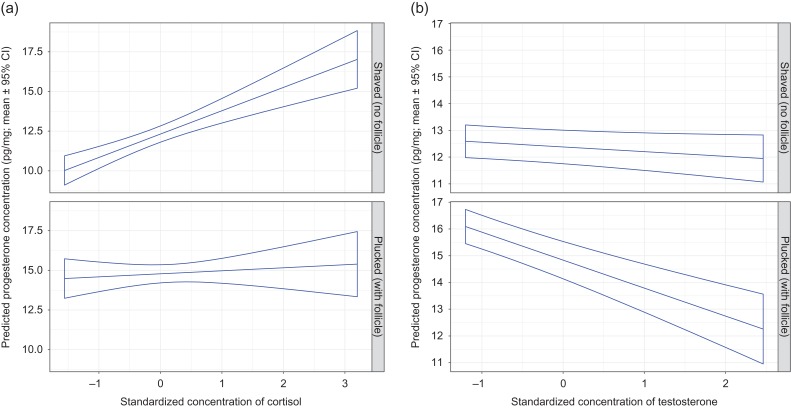
The predicted mean progesterone concentration in the hair of captive adult brown bears in relation to the standardized hair (**a**) cortisol and (**b**) testosterone concentrations, by method of hair collection. The means and 95% confidence intervals were estimated by resampling of data from 94 records using model P1 presented in Table S3a. With shaved samples, the assay was used to determine the progesterone concentration for guard hair shafts only. With plucked samples, progesterone concentrations reflect guard hairs with intact follicles. The analysis was constrained to breeding female bears that were sampled during the hibernation phase on January 4th. Standardized continuous variables in model P1 were set at mean values as follows: testosterone = 0 for panel (a), cortisol = 0 for panel (b), estradiol = 0 for both panels and ordinal day = 1.306 for both panels.

The type of hair sample, shaved or plucked, affected the associations between progesterone and cortisol, and between progesterone and testosterone (Fig. 8). Progesterone increased strongly as cortisol increased in shaved hair samples, but this positive association was considerably weaker in plucked hair samples (Fig. 8a). In contrast, progesterone decreased strongly as testosterone increased in plucked hair samples, but this negative association was less evident in shaved hair samples (Fig. 8b).

#### Estradiol

The fixed effects in our top model (E1) explained ~67% of the variation in estradiol concentrations among bears (Table S4a). The 95% BCI (0.0182–0.2469) for bear identification did not include a value of zero which supported its inclusion as a random intercept in the model. The next ranked model, E2, was not supported by our analysis (∆AIC_C_ = 8.01, *w*_*i*_ = 0.02). Estradiol concentrations were significantly related to testosterone, progesterone and method of hair collection (Table S4b). Further, the association between estradiol and progesterone was affected by method of hair collection.

**Figure 9:**
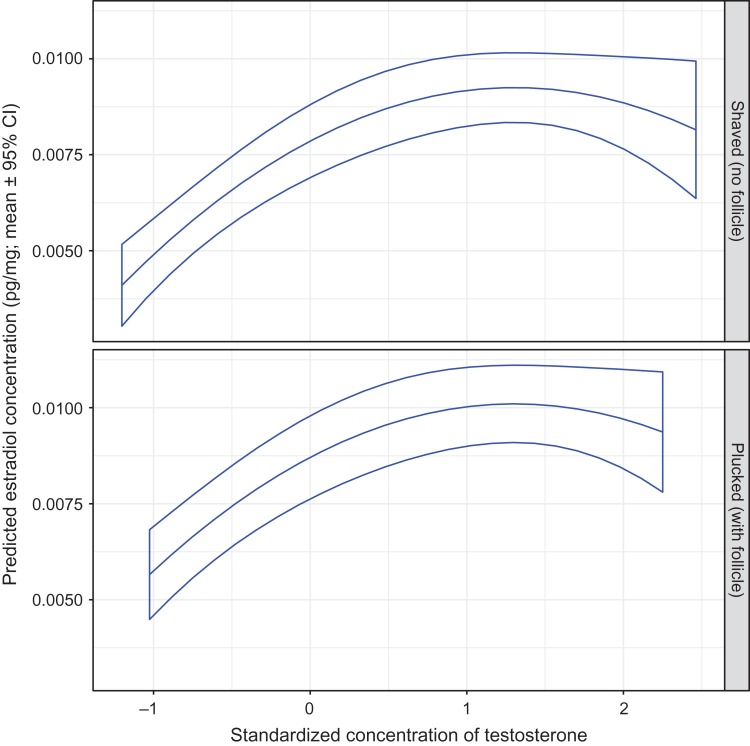
The predicted mean estradiol concentration in the hair of captive adult brown bears in relation to the standardized hair testosterone concentration, and by method of hair collection. The means and 95% confidence intervals were estimated by resampling of data from 94 records using model E1 presented in Table S4a. With shaved samples, the assay was used to determine the testosterone concentration for guard hair shafts only. With plucked samples, testosterone concentrations reflect guard hairs with intact follicles. Standardized continuous variables in model E1 were set at mean values as follows: cortisol = 0 and progesterone = 0.

The hair estradiol concentration in all bears increased as testosterone increased, but the association was curvilinear such that the slope of increase for estradiol diminished as the testosterone concentration increased (Fig. 9). When holding the testosterone level constant, estradiol concentrations tended to be greater in plucked than in shaved hair samples. Although estradiol levels were not significantly associated with ordinal day, estradiol levels did change throughout the year in a similar pattern to what occurred with testosterone (Figs 11 and 12). That is, hair estradiol levels were greatest during pre-breeding based on the analysis of shaved samples (Fig. 12), or at the onset of breeding based on the analysis of plucked samples (Fig. 11). Irrespective of sample type (shaved or plucked) though, estradiol levels decreased throughout the breeding phase and early post-breeding reaching lowest levels by August, and remaining at low levels throughout post-breeding and hibernation phases (Figs 11 and 12).

**Figure 10:**
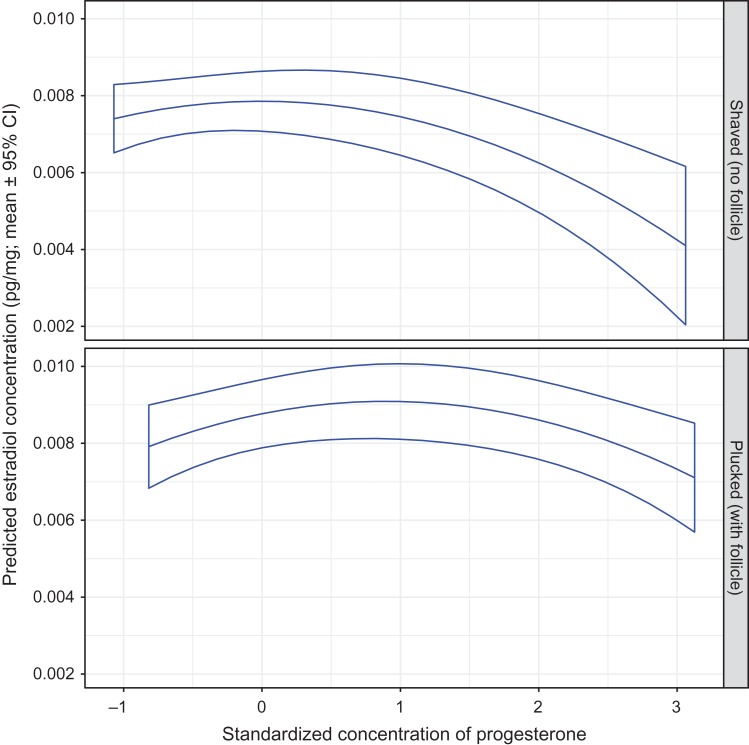
The predicted mean estradiol concentration in the hair of captive adult brown bears in relation to the standardized hair progesterone concentration, and by method of hair collection. The means and 95% confidence intervals were estimated by resampling of data from 94 records using model E1 presented in Table S4a. With shaved samples, the assay was used to determine the progesterone concentration for guard hair shafts only. With plucked samples, progesterone concentrations reflect guard hairs with intact follicles. Standardized continuous variables in model E1 were set at mean values as follows: cortisol = 0 and testosterone = 0.

The hair estradiol concentration decreased as progesterone increased (Fig. 10). As with testosterone, the association with progesterone was curvilinear, but negative, such that the slope of decrease for estradiol became steeper as progesterone levels increased. This pattern of change in slope was most evident in hair samples collected by shaving.

**Figure 11:**
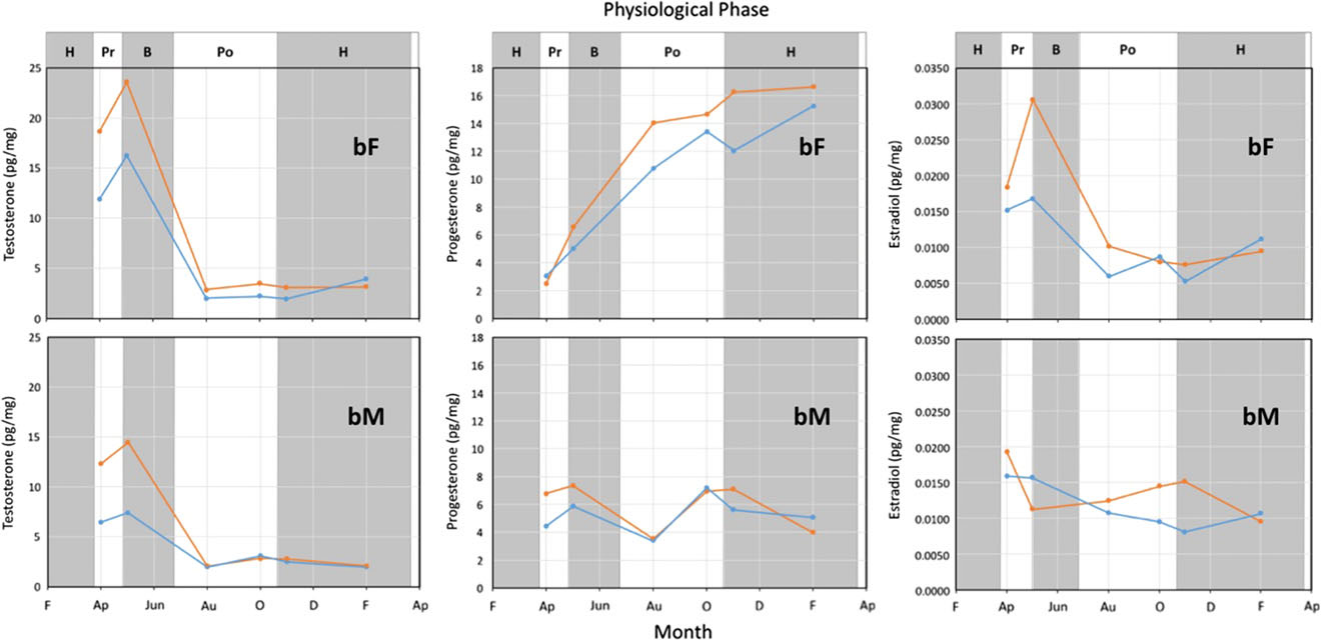
Reproductive hormone concentrations measured in plucked hair samples (includes follicles) collected from two female (bF) and two male (bM) captive brown bears that bred successfully in May 2014. The females each gave birth to two cubs in early to mid-January 2015. The physiological phases are hibernation (H), pre-breeding (Pr), breeding (B) and post-breeding (Po).

**Figure 12:**
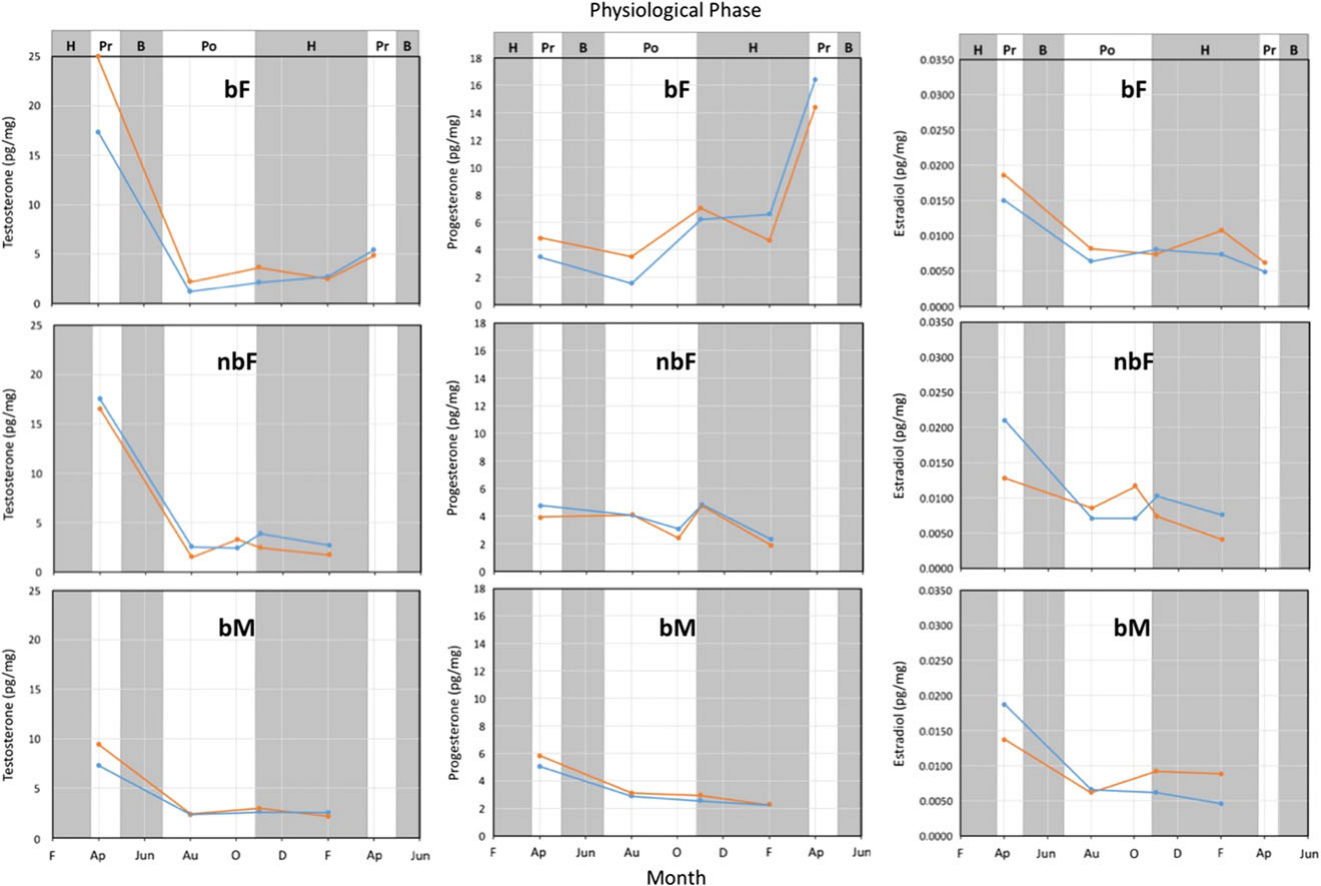
Reproductive hormone concentrations measured in shaved hair samples (does not include follicles) collected from six captive brown bears from April 2014 to April 2015. Two female (bF) and two male bears (bM) that bred successfully in May 2014, and the females each gave birth to two cubs in early to mid-January 2015. Two non-breeding females (nbF) were administered megestrol acetate as a method of birth control from mid-April to mid-June. The physiological phases are hibernation (H), pre-breeding (Pr), breeding (B) and post-breeding (Po).

## Discussion

In this study, we first established the reliability and practicality of enzyme immunoassay-based procedures to measure the concentrations of three steroid hormones, testosterone, progesterone and estradiol, in hair samples collected from brown bears. We then determined, through the analysis of hormone concentrations in serial hair samples collected from captive adult brown bears, that reproductive hormone levels: (i) were correlated with each other, as well as with the hair cortisol concentration; (ii) differed, but were correlated, between hair samples that were collected by shaving vs. samples collected by plucking; (iii) varied by sex; and (iv) varied by reproductive state within females. Further, within individual bears, hormone levels also changed throughout the year in step with different reproductive events.

All hormone concentrations determined in this study were based on the measurement of unbound (free) hormone in brown bear guard hairs. Although we could have measured the same hormones in the undercoat hair, we intentionally selected and removed guard hairs from collected hair samples for laboratory analysis because of reasons provided by Macbeth *et al*. (2010), which include a longer window of time for the annual growth of guard hair vs. undercoat hair, less variability in hormone concentrations between body regions at the same point in time, and the relative ease of cleaning and grinding guard hair. We required ~125 mg of guard hair, which equates to 125–250 individual guard hairs, to reliably measure, in duplicate, the concentrations of unbound testosterone, progesterone, estradiol and cortisol contained within hairs. Expressed another way, we required 25 mg of guard hair per hormone except for estradiol which required 50 mg. This amount, however, far exceeded what is typically snagged by a single barb of barbed wire which was determined by Macbeth *et al*. (2010) to be ≤30 mg. Given this limitation, we recommend that future studies ascertain the ease and reliability of using mixed (guard and undercoat) hair samples and, in the case of non-invasive sampling, that hair collection techniques are chosen to maximize the amount of hair collected per animal, e.g. multiple strands of barbed wire and/or use of wire with closely-spaced (5 cm) barbs.

The assay performance standards for testosterone and progesterone were good in that parallelism was clearly evident (*R*^2^ > 0.975), the lower LOD were well below the minimum values recorded for all samples used in this study, the variation (precision) in hormone measurements for the same sample both within and between assays was acceptable (<15%; FDA, 2001), and extraction efficiencies (hormone recoveries) were consistent and precise. The assay performance standards for estradiol, however, were not as good based on two findings. First, although all values were above the lower detection limit, many of the estradiol values were at the low end of the dilution curve for the assay where accuracy and precision were generally less than at the mid-range of the curve. Second, the extraction efficiency for estradiol was unrealistically high (>160%) and inconsistent. Because of these findings, we believe that the estradiol values were less accurate and/or precise than the testosterone and progesterone values reported for this study.

In the context of this study, the measurement of estradiol did not better enable us to differentiate between sex and reproductive states at different times of the year, which was our fourth research objective. It did, however, assist us in meeting our second and third research objectives, that is to gain insight into the inter-correlations among steroid hormones in hair, and to evaluate if the method of hair collection affected the resultant concentrations of reproductive hair hormones. Further, the measurement of estradiol may also prove useful in expanded studies, now underway, to establish if reproductive hormone concentrations in hair samples collected from free-ranging bears can be used to discriminate between age classes (immature vs. mature), as well as sex and reproductive classes. Taken together at this time, we see more value in trying to improve the accuracy and precision of measuring estradiol rather than dropping it from the collection of steroid hormones that we are measuring in hair. In this regard, the application of mass spectrometry methods, which are considered to be the ‘gold standard’ for hair analysis (Gow *et al*., 2010), instead of enzyme-linked immunosorbent assays, as were used for this study, may provide a more accurate and precise approach, with greater sensitivity and specificity, for the measurement of steroid hormones in hair (Gao *et al*., 2013; Kapoor *et al*., 2016; Weisser *et al*., 2016).

The significant associations among the different reproductive hormones and cortisol found in this study were hoped for given that these organic compounds, as steroid hormones, are all derived from cholesterol, and are formed along common biosynthetic pathways (Boron and Boulpaep, 2016). It is outside the scope of this study for us to explain or speculate on the physiological basis for many of these associations, as shown in Figs 3–10. Nonetheless, they suggest that the integration of reproductive hormones and cortisol in hair was unlikely to have been haphazard and more likely mirrored underlying physiological processes. These figures were included in this report largely to serve as an impetus for future studies.

We do not know the primary source of reproductive hormones (i.e. systemic blood flow or local skin production) or how they are sequestered into brown bear hair. Nonetheless, a few observations from this study suggest that hair hormone concentrations were influenced by more than just passive diffusion from the systemic blood circulation. One observation was the change we observed in the hair hormone levels of some bears during the quiescent (telogen) phase of hair development, which essentially spans the physiological phases of hibernation, pre-breeding, and the first 3 weeks of breeding, i.e. from late October to mid-May. During the onset of quiescence, hair growth ceases and blood flow to follicles is terminated (Stenn and Paus, 2001), so passive diffusion of hormones from the vascular supply after this point seems unlikely. Nonetheless, we observed 2–4-fold increases in testosterone (e.g. from 2.70 to 5.45 pg/mg) and progesterone concentrations (e.g. from 4.70 to 14.39 pg/mg) in individual bears between samples collected sequentially during hibernation followed by pre-breeding in the same quiescent hair phase. Estradiol and cortisol concentrations also changed over this period, but the direction of change was inconsistent and the magnitude of change tended to be smaller. The other observation was that the concentrations of progesterone, and the association between progesterone and testosterone levels, differed markedly between plucked and shaved hair samples. Estradiol concentrations also differed between the two types of samples, generally being higher in plucked samples, but the differences were not as evident as with progesterone. Overall, this suggests that the hair follicle functions as more than a simple conduit for hormones to diffuse from the blood circulation into growing hairs. Indeed, ample research over the past 10 years has demonstrated that hair follicles or, more broadly, skin cells contain the full suite of substrates and enzymes required for the biosynthesis of reproductive steroid hormones, as well as cortisol and vitamin D (Reichrath, 2007; Slominski *et al*., 2007, 2013; Zouboulis, 2009; Inoue *et al*., 2012; Reichrath *et al*., 2016). Further, these steroids of skin origin affect a wide range of functions which include hair growth (Foitzik *et al*., 2006; Ohnemus *et al*., 2006), skin growth and maintenance (Inoue *et al*., 2012), and local immunity (Slominski *et al*., 2013). This does not imply, however, that hormones measured in hair reflect local production only. Like many organs, skin is controlled both through local (paracrine, autocrine and intracrine) and distant (endocrine) molecular signals (Nikolakis *et al*., 2016). So, steroids sequestered in hair are likely derived from various steroidogenic organs in proportions that are variable and dictated by the relative influences of multiple, synchronized physiological processes.

From the standpoint of using hair reproductive hormone concentrations as physiological indicators in the context of wildlife conservation and research, it is clear that the method of hair collection (plucking vs. shaving) is an important consideration when interpreting or comparing results. In this regard, our research efforts to date may have fallen short when evaluating the full potential for hair hormone analyses to support wildlife conservation efforts. If hair follicles are, indeed, mini-organs as dubbed in the human physiology and pathology literature (Plikus *et al*., 2015; Chen *et al*., 2016), then hair hormone studies in the context of wildlife conservation may be more insightful when based on the analysis of full hairs that include follicles. Still, much of the research reported to date has been based on analyses of shaved or cut hair (Bryan *et al*., 2014a, b; Terwissen *et al*., 2014; Mislan *et al*., 2016) or intact hair from which follicles were manually removed (Carlitz *et al*., 2016). In this study, the concentrations of all hormones, except testosterone, tended to be greater in guard hairs plucked vs. shaved from brown bears. However, we estimate that the follicle probably accounted for no more than 4% of the length of a typical guard hair (e.g. 4 mm follicle for a 100 mm hair (Elgmork and Riiser, 1991)), and perhaps a slightly greater percentage of the hair mass given its greater diameter relative to the hair shaft (Otberg *et al*., 2004). And yet, the quantity of hormone contained within the follicle was often large enough to measurably affect the hormone concentration of the entire hair. Although the hair follicle is supplied by peri- and inter-follicular blood vessels (Stenn and Paus, 2001), many of which are developed by the hair follicles themselves to form a follicle-linking network (Amoh *et al*., 2004, 2005), the follicle itself does not have internalized blood capillaries. So, the high concentration of hormone in the follicle is unlikely to be a consequence of blood contamination (i.e. contained blood), but may instead reflect synthesis (Ohnemus *et al*., 2006; Zouboulis, 2009) and long-term storage (Lademann *et al*., 2006) by follicular cells. Undoubtedly, more study is needed to determine if the inclusion of follicles in the laboratory analysis of hair samples may enable a more accurate assessment of physiological state. In addition, we need to understand the relative time-frame reflected by hormone concentrations measured in hair samples analyzed with follicles intact. To date, hormone measurements from shaved hair samples, or from samples where the follicles have been manually trimmed, have been described as integrated (long-term) measurements reflecting the average concentration over the time that the hair has been growing (Macbeth *et al*., 2010). Although we do not know the time frame reflected by hormone levels in the follicle, the hormone dynamics presented in Fig. 11, particularly the progesterone dynamics in breeding females, would suggest it is substantially longer than the point-in-time picture provided by the measurement of hormone levels in blood.

While the hair reproductive hormone profile may be affected by physiological processes other than reproduction, our findings suggest that reproduction does in fact play a major role in influencing the hair reproductive hormone profile. Changes in hair hormone concentrations over time, and in conjunction with key reproductive events, were similar to what has been reported by others studying hormonal changes in the blood serum of brown bears. This includes (i) testosterone levels in male bears increase during pre-breeding, peak during breeding and then recede during the latter half of breeding (Tsubota and Kanagawa, 1989); and (ii) progesterone levels of pregnant females rise in a sustained manner following breeding (Tsubota *et al*., 1992). Surprisingly though, reports concerning reproductive hormone changes in the blood serum of brown bears are sparse, and too few to allow us to fully compare profiles (i.e. three hormones × sex and reproductive state × physiological phase) between hair and serum. In fact, our study is the first to our knowledge to document the full reproductive hormones profiles of adult female and male brown bears. Still, blood serum reproductive hormone profiles have been reported for other species of Ursidae, including black (*U. americanus*) and polar (*U. maritimus*) bears (Palmer *et al*., 1988; Hellgren *et al*., 1990; Tsubota *et al*., 1998), and hormonal responses to key reproductive events are relatively similar to what we observed in brown bears.

We also found in this study that hair testosterone levels tended to be greater in females than males. Surprisingly, however, no attention has been given to the role(s) of testosterone in female bears as underscored by the many studies of reproduction, or reproductive hormones, in a variety of bear species over the past 40 years in which testosterone dynamics or differences are studied in males only, and estradiol and progesterone are studied in females only. Even with research involving humans, testosterone is traditionally associated with, and studied in males, whereas studies of females have focused on the effects of progesterone and estradiol (Arnon *et al*., 2016). Our findings from this study contrast to some degree with those of Bryan *et al*. (2013) who, in one study, found that hair testosterone levels in brown bears were greater in males than females but, in another study (Bryan *et al*., 2014a), found that hair testosterone levels were similar between sexes in both brown and black (*U. americanus*) bears. Given recent attention to the potential adaptive consequences of maternal testosterone levels on offspring attributes in mammals, including sex ratio (Grant and Chamley, 2010; Edwards *et al*., 2016) and behaviour (Dloniak *et al*., 2006), the measurement of hair testosterone levels in female bears could prove useful to better understanding ursid life histories.

Although concurrent concentrations of multiple reproductive hormones in hair have been reported previously for various wild mammals (see, e.g. hair testosterone and progesterone in wolves (*Canis lupus*; Bryan *et al*. 2014b), and hair testosterone and estradiol in ring-tailed lemurs (*Lemur catta*; Tennenhouse *et al*., 2016), few studies have evaluated these measurements from the standpoint of their potential value in augmenting non-invasive genetic sampling (Schwartz and Monfort, 2008). An exception is the study by Terwissen *et al*. (2014) where reproductive hormone profiles (testosterone, progesterone, estradiol) of hair samples collected from 73 Canada lynx (*Lynx canadensis*) pelts were assessed for their utility to differentiate between age and sex. The authors reported limited success using the hair estradiol-to-progesterone ratio to differentiate sexes, but not age classes, and concluded that further refinement and validation of hair hormone analyses by enzyme immunoassay would be required before this technique could be applied broadly. In contrast, our findings suggest the potential for hair reproductive hormone levels to support non-invasive genetic sampling by enabling differentiation between sex and reproductive state of brown bears is strong. We recognize, however, that our findings were constrained by the small number of bears, the fact that all study animals were adults and the fact that all study animals were captive. Consequently, we are expanding our current studies to establish if reproductive hormone concentrations in hair samples collected from free-ranging bears under a variety of conditions (i.e. differences in body condition, different types of human contact and different capture histories) can be used to discriminate between age classes (immature vs. mature), as well as sex and reproductive classes.

## Supplementary Material

Supplementary Data
