# Supplementary material for: The quantification of reproductive hormones in the hair of captive adult brown bears and their application as indicators of sex and reproductive state
Source: Conserv Physiol. 2017 Jun 1;5(1):cox032. doi: 10.1093/conphys/cox032 (PMC5452076; doi:10.1093/conphys/cox032)
Supplement: Supplementary Data [file CONPHYS-2017-001_revised_supp_16Apr2017.docx]

Table S1. Classification of 94 hair samples into categories by sex and reproductive class and by physiological phase. Data were complete for all categories, i.e., no missing values.

|  | Sex and reproductive class [Number of individuals] | | |  |
| --- | --- | --- | --- | --- |
| Physiological Phase | Breeding female [2] | Non-breeding female [4] | Breeding male [2] | Total |
| Hibernation | 14 | 16 | 15 | 45 |
| Pre-breeding | 6 | 5 | 4 | 15 |
| Breeding | 2 | 6 | 2 | 10 |
| Post-breeding | 6 | 14 | 4 | 24 |
| Total | 28 | 41 | 25 | 94 |

Table S2a. Comparison of candidate models^a^ by sample-size–adjusted Akaike information criterion (AIC_C_)^b^ to predict the concentration of testosterone in 94 hair samples collected from captive adult brown bears. Bold typeface denotes models for which ∆AIC_C_ ≤ 2.00. All models include an intercept and random effect^c^. Model T10 is an intercept only (null) model.

| Model | Hormone | Biology | Time | Method | Interactions | K | AIC_C_ | ∆AIC_C_ | *w_i_* | *R^2^_LR_* |
| --- | --- | --- | --- | --- | --- | --- | --- | --- | --- | --- |
| **T1** | **cort + estra + estra^2^** | **src** | **d + d^2^ + d^3^** |  | **src × estra^2^, src × d** | **15** | **446.0** | **0.00** | **0.76** | **0.823** |
| T2 | cort + estra + estra^2^ | src | d + d^2^ + d^3^ | f | src × estra^2^, src × d | 16 | 448.3 | 2.35 | 0.24 | 0.824 |
| T3 | cort + estra + estra^2^ |  | d + d^2^ + d^3^ |  | src × d | 9 | 467.9 | 21.96 | 0.00 | 0.731 |
| T4 | cort + estra + estra^2^ | src |  |  | src × estra^2^ | 10 | 473.5 | 27.59 | 0.00 | 0.722 |
| T5 | cort + estra + estra^2^ | src |  | f | src × estra^2^ | 11 | 475.6 | 29.62 | 0.00 | 0.723 |
| T6 | cort + estra + estra^2^ |  |  | f |  | 7 | 479.1 | 33.18 | 0.00 | 0.679 |
| T7 | cort + estra + estra^2^ |  |  |  |  | 6 | 480.5 | 34.55 | 0.00 | 0.666 |
| T8 |  |  | d + d^2^ + d^3^ |  |  | 6 | 544.5 | 98.52 | 0.00 | 0.328 |
| T9 |  | src |  |  |  | 5 | 564.8 | 118.9 | 0.00 | 0.140 |
| T10 |  |  |  |  |  | 3 | 574.3 | 128.33 | 0.00 | - |
| T11 |  |  |  | f |  | 4 | 576.4 | 130.45 | 0.00 | 0.001 |

^a^ Variables include cortisol (cort), estradiol (estra, estra^2^), sex and reproductive class (src), ordinal day (d, d^2^, d^3^), and follicle (f).

^b^ Statistics include number of estimable parameters in model (*K*), sample-size–adjusted Akaike information criterion (AIC_C_), difference in AIC_C_ between top model and model *i* (∆AIC_C_), Akaike weight for model *i* (*w_i_*), and a coefficient of determination based on the likelihood-ratio test (*R^2^_LR_*).

^c^ Bear identification is included as a random effect (intercept) in all models.

Table S2b. Standardized coefficients, standard errors (SE), and 95% Bayesian credible intervals (BCI) for the estimable parameters in the top predictive model (∆AIC_C_ = 0.00) from Table S2a. The response variable is the standardized concentration of testosterone in 94 hair samples collected from captive adult brown bears. Bold typeface denotes 95% BCI that do not include a value of zero.

| Estimable parameter^a^ | Standardized coefficient | SE | 2.5% quantile | 97.5% quantile |
| --- | --- | --- | --- | --- |
| **intercept** | **1.4340** | **0.2802** | **0.8457** | **1.9495** |
| **random intercept^b^** | **0.4130** | **0.2152** | **0.1509** | **0.9526** |
| **cort** | **-0.1578** | **0.0619** | **-0.2798** | **-0.0368** |
| **estra** | **0.5849** | **0.0518** | **0.4832** | **0.6858** |
| estra^2^ | -0.0744 | 0.0406 | -0.1507 | 0.0085 |
| **src (non-breeding female)** | **0.8561** | **0.2231** | **0.4307** | **1.3006** |
| src (breeding male) | -0.1406 | 0.4343 | -0.9641 | 0.7694 |
| d | 0.0179 | 0.1401 | -0.2611 | 0.2859 |
| **d^2^** | **0.1479** | **0.0473** | **0.0557** | **0.2417** |
| **d^3^** | **-0.1318** | **0.0532** | **-0.2344** | **-0.0264** |
| estra^2^ × src (non-breeding female) | -0.1266 | 0.0754 | -0.2769 | 0.0193 |
| **estra^2^ × src (breeding male)** | **0.3133** | **0.1339** | **0.0506** | **0.5743** |
| d × src (non-breeding female) | 0.1793 | 0.1035 | -0.0201 | 0.3850 |
| d × src (breeding male) | 0.0362 | 0.1068 | -0.1684 | 0.2455 |

^a^ Estimable parameters included the intercept, cortisol (cort), estradiol (estra, estra^2^), sex and reproductive class (src), and ordinal day (d, d^2^, d^3^).

^b^ Bear identification was included as a random effect (intercept).

Table S3a. Comparison of candidate models^a^ by sample-size–adjusted Akaike information criterion (AIC_C_)^b^ to predict the concentration of progesterone in 94 hair samples collected from captive adult brown bears. Bold typeface denotes models for which ∆AIC_C_ ≤ 2.00. All models include an intercept and random effect^c^. Model P11 is an intercept only (null) model.

| Model | Hormone | Biology | Time | Method | Interactions | *K* | AIC_C_ | ∆AIC_C_ | *w_i_* | *R^2^_LR_* |
| --- | --- | --- | --- | --- | --- | --- | --- | --- | --- | --- |
| **P1** | **cort + test** | **src** | **d + d^2^ + d^3^** | **f** | **(src × cort) + (src × d) + (f × cort) + (f × test) + (f × d)** | **18** | **367.5** | **0.00** | **0.98** | **0.662** |
| P2 | cort + test | src |  | f | (src × cort) + (f × cort) + (f × test) | 12 | 375.0 | 7.49 | 0.02 | 0.554 |
| P3 | cort + test |  | d + d^2^ + d^3^ |  | (src × cort) + (src × d) | 14 | 383.5 | 15.98 | 0.00 | 0.539 |
| P4 | cort + test | src |  |  | (src × cort) | 9 | 389.1 | 21.62 | 0.00 | 0.432 |
| P5 | cort + test |  |  | f | (f × cort) + (f × test) | 8 | 395.8 | 28.32 | 0.00 | 0.371 |
| P6 |  | src |  |  |  | 5 | 405.9 | 38.46 | 0.00 | 0.240 |
| P7 | cort + test |  | d + d^2^ + d^3^ |  |  | 8 | 412.1 | 44.63 | 0.00 | 0.247 |
| P8 |  |  |  | f |  | 4 | 418.9 | 51.39 | 0.00 | 0.102 |
| P9 | cort + test |  |  |  |  | 5 | 420.9 | 53.45 | 0.00 | 0.103 |
| P10 |  |  | d + d^2^ + d^3^ |  |  | 6 | 423.1 | 55.57 | 0.00 | 0.105 |
| P11 |  |  |  |  |  | 3 | 426.4 | 58.96 | 0.00 | - |

^a^ Variables include cortisol (cort), testosterone (test), sex and reproductive class (src), ordinal day (d, d^2^, d^3^), and follicle (f).

^b^ Statistics include number of estimable parameters in model (*K*), sample-size–adjusted Akaike information criterion (AIC_C_), difference in AIC_C_ between top model and model *i* (∆AIC_C_), and Akaike weight for model *i* (*w_i_*).

^c^ Bear identification is included as a random effect (intercept) in all models.

Table S3b. Standardized coefficients, standard errors (SE), and 95% Bayesian credible intervals (BCI) for the estimable parameters in the top predictive model (∆AIC_C_ = 0.00) from Table S3a. The response variable is the standardized concentration of progesterone in 94 hair samples collected from captive adult brown bears. Bold typeface denotes 95% BCI that do not include a value of zero.

| Estimable parameter^a^ | Standardized coefficient | SE | 2.5% quantile | 97.5% quantile |
| --- | --- | --- | --- | --- |
| **intercept** | **1.7144** | **0.1888** | **1.3655** | **2.1220** |
| **random intercept^b^** | **0.1533** | **0.1242** | **0.0177** | **0.4783** |
| **cort** | **0.3843** | **0.1940** | **0.0026** | **0.7667** |
| test | -0.0448 | 0.0619 | -0.1659 | 0.0775 |
| **src (non-breeding female)** | **-0.6072** | **0.1954** | **-1.0447** | **-0.2586** |
| **src (breeding male)** | **-0.6491** | **0.2262** | **-1.1438** | **-0.2389** |
| **d** | **0.4017** | **0.1476** | **0.1107** | **0.6962** |
| **d^2^** | **0.0983** | **0.0493** | **0.0033** | **0.1974** |
| **d^3^** | **-0.1959** | **0.0539** | **-0.3029** | **-0.0896** |
| **f (yes)** | **0.4302** | **0.1072** | **0.2213** | **0.6423** |
| cort × src (non-breeding female) | -0.1689 | 0.1997 | -0.5580 | 0.2206 |
| cort × src (breeding male) | -0.3303 | 0.2290 | -0.7880 | 0.1148 |
| d × src (non-breeding female) | -0.1088 | 0.1131 | -0.3322 | 0.1081 |
| **d × src (breeding male)** | **-0.2443** | **0.1143** | **-0.4676** | **-0.0172** |
| **cort × f (yes)** | **-0.3460** | **0.1333** | **-0.6056** | **-0.0796** |
| **test × f (yes)** | **-0.2409** | **0.1172** | **-0.4722** | **-0.0121** |
| d × f (yes) | 0.1752 | 0.1293 | -0.0780 | 0.4260 |

^a^ Estimable parameters included the intercept, cortisol (cort), testosterone (test), sex and reproductive class (src), follicle (f), and ordinal day (d, d^2^, d^3^).

^b^ Bear identification was included as a random effect (intercept).

Table S4a. Comparison of candidate models^a^ by sample-size–adjusted Akaike information criterion (AIC_C_)^b^ to predict the concentration of estradiol in 94 hair samples collected from captive adult brown bears. Bold typeface denotes models for which ∆AIC_C_ ≤ 2.00. All models include an intercept and random effect^c^. Model E11 is an intercept only (null) model.

| Model | Hormone | Biology | Time | Method | Interactions | *K* | AIC_C_ | ∆AIC_C_ | *w_i_* | *R^2^_LR_* |
| --- | --- | --- | --- | --- | --- | --- | --- | --- | --- | --- |
| **E1** | **cort + cort^2^ +test + test^2^ + prog + prog^2^** |  |  | **f** | **(f × prog)** | **11** | **-786.9** | **0.00** | **0.96** | **0.674** |
| E2 | cort + cort^2^ +test + test^2^ + prog + prog^2^ |  |  |  |  | 9 | -778.9 | 8.01 | 0.02 | 0.624 |
| E3 | cort + cort^2^ +test + test^2^ + prog + prog^2^ | src |  | f | (src × cort) + (src × test) + (src × test^2^) + (src × prog) + (src × prog^2^) + (f × prog) | 23 | -778.7 | 8.26 | 0.00 | 0.761 |
| E4 | cort + cort^2^ +test + test^2^ + prog + prog^2^ |  | d |  |  | 10 | -776.4 | 10.52 | 0.00 | 0.624 |
| E5 | cort + cort^2^ +test + test^2^ + prog + prog^2^ | src | d | f | (src × cort) + (src × test) + (src × test^2^) + (src × prog) + (src × prog^2^) + (f × prog) + (src × d) | 26 | -773.6 | 13.36 | 0.00 | 0.777 |
| E6 | cort + cort^2^ +test + test^2^ + prog + prog^2^ | src | d |  | (src × cort) + (src × test) + (src × test^2^) + (src × prog) + (src × prog^2^) + (src × d) | 24 | -771.7 | 15.20 | 0.00 | 0.753 |
| E7 | cort + cort^2^ +test + test^2^ + prog + prog^2^ | src |  |  | (src × cort) + (src × test) + (src × test^2^) + (src × prog) + (src × prog^2^) | 21 | -770.1 | 16.82 | 0.00 | 0.717 |
| E8 |  |  | d |  |  | 4 | -715.9 | 71.00 | 0.00 | 0.153 |
| E9 |  | src |  |  |  | 5 | -706.5 | 80.45 | 0.00 | 0.084 |
| E10 |  |  |  | f |  | 4 | -702.8 | 84.11 | 0.00 | 0.024 |
| E11 |  |  |  |  |  | 3 | -702.8 | 84.13 | 0.00 | - |

^a^ Variables include cortisol (cort, cort^2^), testosterone (test, test^2^), progesterone (prog, prog^2^), sex and reproductive class (src), ordinal day (d), and follicle (f).

^b^ Statistics include number of estimable parameters in model (*K*), sample-size–adjusted Akaike information criterion (AIC_C_), difference in AIC_C_ between top model and model *i* (∆AIC_C_), and Akaike weight for model *i* (*w_i_*).

^c^ Bear identification is included as a random effect (intercept) in all models.

Table S4b. Standardized coefficients, standard errors (SE), and 95% Bayesian credible intervals (BCI) for the estimable parameters in the top predictive model (∆AIC_C_ = 0.00) from Table S4a. The response variable is the standardized concentration of estradiol in 94 hair samples collected from captive adult brown bears. Bold typeface denotes 95% BCI that do not include a value of zero.

| Estimable parameter^a^ | Standardized coefficient | SE | 2.5% quantile | 97.5% quantile |
| --- | --- | --- | --- | --- |
| **intercept** | **-4.2951** | **0.0814** | **-4.4621** | **-4.1370** |
| **random intercept^b^** | **0.1032** | **0.0594** | **0.0182** | **0.2469** |
| cort | 0.0127 | 0.0365 | -0.0578 | 0.0832 |
| cort^2^ | 0.0341 | 0.0209 | -0.0071 | 0.0754 |
| **test** | **0.4045** | **0.0432** | **0.3149** | **0.4869** |
| **test^2^** | **-0.1534** | **0.0378** | **-0.2254** | **-0.0715** |
| prog | -0.0228 | 0.0666 | -0.1596 | 0.1052 |
| **prog^2^** | **-0.0675** | **0.0289** | **-0.1228** | **-0.0095** |
| **f (yes)** | **0.1755** | **0.0666** | **0.0395** | **0.3011** |
| **prog × f (yes)** | **0.1444** | **0.0640** | **0.0192** | **0.2716** |

^a^ Estimable parameters included the intercept, cortisol (cort, cort^2^), testosterone (test, test^2^), progesterone (prog, prog^2^), and follicle (f).

^b^ Bear identification was included as a random effect (intercept).

a) b)

c) d)

Figure S1. Markov Chain Monte Carlo (MCMC) diagnostics for the top model (T1) in Table 3 included a visual assessment of the a) mixing of chains and b) posterior distributions for each parameter. Validation of T1 included a visual assessment of the c) scatterplot of Pearson residuals versus fitted values and d) quantile-quantile (q-q) plot of the residuals. Parameters are beta[1] – intercept, beta[2] – cort, beta[3] – estra, beta[4] – src (non-breeding female), beta[5] – src (breeding male), beta[6] – estra^2^, beta[7] – d, beta[8] – d^2^, beta[9] – d^3^, beta[10] – estra^2^ × src (non-breeding female), beta[11] – estra^2^ × src (breeding male), beta[12] – d × src (non-breeding female), beta[13] – d × src (breeding male), sigma.ri1 – random intercept, and tau – residual.

a) b)

c) d)

Figure S2. Markov Chain Monte Carlo (MCMC) diagnostics for the top model (P1) in Table 4 included a visual assessment of the a) mixing of chains and b) posterior distributions for each parameter. Validation of P1 included a visual assessment of the c) scatterplot of Pearson residuals versus fitted values and d) quantile-quantile (q-q) plot of the residuals. Parameters are beta[1] – intercept, beta[2] – cort, beta[3] – test, beta[4] – src (non-breeding female), beta[5] – src (breeding male), beta[6] – d, beta[7] – d^2^, beta[8] – d^3^, beta[9] – f (yes), beta[10] – cort × src (non-breeding female), beta[11] – cort × src (breeding male), beta[12] – d × src (non-breeding female), beta[13] – d × src (breeding male), beta[14] – cort × f (yes), beta[15] – test × f (yes), beta[16] – d × f (yes), sigma.ri1 – random intercept, and tau – residual.

a) b)

c) d)

Figure S3. Markov Chain Monte Carlo (MCMC) diagnostics for the top model (E1) in Table 5 included a visual assessment of the a) mixing of chains and b) posterior distributions for each parameter. Validation of E1 included a visual assessment of the c) scatterplot of Pearson residuals versus fitted values and d) quantile-quantile (q-q) plot of the residuals. Parameters are beta[1] – intercept, beta[2] – cort, beta[3] – cort^2^, beta[4] – test, beta[5] – test^2^, beta[6] – prog, beta[7] – prog^2^, beta[8] – f (yes), beta[9] – prog × f (yes), sigma.ri1 – random intercept, and tau – residual.
